# A selective dual quenching sensor (EY/BG@CDs) for simultaneous monitoring of gentamicin and ketorolac levels in plasma: a highly efficient platform that caters to the needs of therapeutic drug monitoring†

**DOI:** 10.1039/d3ra04894b

**Published:** 2023-10-03

**Authors:** Khalid Alhazzani, Ahmed Z. Alanazi, Aya M. Mostafa, James Barker, Mohamed M. El-Wekil, Al-Montaser Bellah H. Ali

**Affiliations:** a Department of Pharmacology and Toxicology, College of Pharmacy, King Saud University Riyadh Saudi Arabia; b School of Life Sciences, Pharmacy, and Chemistry, Kingston University Kingston-upon-Thames London KT1 2EE UK; c Department of Pharmaceutical Analytical Chemistry, Faculty of Pharmacy, Assiut University Assiut Egypt Almontaser_bellah@aun.edu.eg

## Abstract

This research work introduces a novel sensor that utilizes two fluorophores to enable simultaneous monitoring of gentamicin sulphate (GNT) and ketorolac tromethamine (KET). The innovative sensor is composed of carbon dots (CDs) derived from black grapes (BG) and eosin Y (EY) dye. The interaction between the studied drugs and EY/BG@CDs sensor components allows for their simultaneous detection where GNT quenches the fluorescence of EY at 535 nm without affecting the fluorescence of CDs, while KET quenches the fluorescence of BG@CDs at 385 nm without impacting EY fluorescence. The BG@CDs probe was successfully characterized using various techniques such as absorption spectrophotometry, spectrofluorimetry, TEM imaging, infrared spectroscopic analysis, and XRD analysis. The suggested methodology was observed to be highly sensitive for the simultaneous determination of GNT and KET in their spiked rabbit plasma samples, with wide linear ranges and low limit of detection (LOD) values. The studied drugs were extracted using a highly selective extraction method involving protein precipitation followed by mixed mode solid phase extraction using an Oasis WCX cartridge. The simultaneous determination of GNT and KET is essential due to the potential interactions between the studied drugs. Therefore, this analysis can be used to evaluate the necessity of dose monitoring and the potential adverse effects of co-administration of these drugs.

## Abstract 2

This research work introduces a novel sensor that utilizes two fluorophores to enable simultaneous monitoring of gentamicin sulphate (GNT) and ketorolac tromethamine (KET).

## Graphical abstract

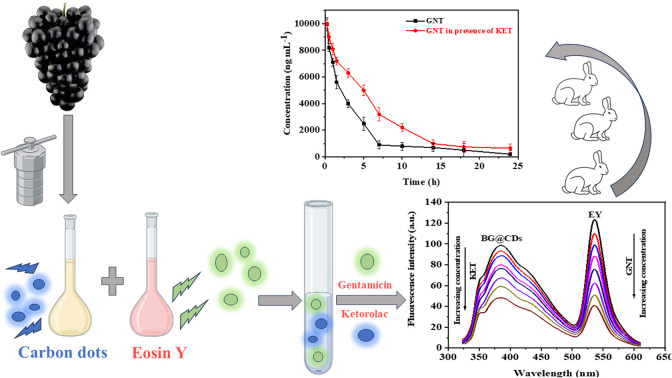

## 1. Introduction

Pharmacokinetic drug–drug interactions related to administration of nonsteroidal anti-inflammatory drugs (NSAIDs) such as ketorolac tromethamine (KET) with aminoglycoside antibiotics such as gentamicin sulfate (GNT) are quite common to patients suffering from severe localized infections.^1,2^ While GNT is a treatment for bacterial infections induced by Gram-negative bacteria, KET is used to manage moderate to severe pain.^3^ It is worth mentioning that the simultaneous use of KET and GNT may increase the risk of renal toxicity, as both drugs can independently affect renal function.^4^ Since GNT is not metabolized, the levels of the drug in plasma and its half-life are reliant on the renal function.^5^ NSAIDs have been shown to cause various renal function abnormalities, and NSAID-induced nephrotoxicity may interfere with the pharmacokinetics of GNT, resulting in the accumulation of GNT and, in turn, severe nephrotoxicity.^6^ This is because KET can decrease the amount of GNT eliminated from the body by the kidneys, leading to higher levels of GNT in the bloodstream and an increased risk of kidney damage. Healthcare providers should closely monitor renal function and consider potential drug interactions when prescribing these medications together. In order to validate this assumption, we determined GNT concentration in rabbits' plasma in the presence of KET to investigate how the pharmacokinetics of GNT were affected by coadministration of KET.

A range of methods are available for determining GNT and KET, including high-performance liquid chromatography (HPLC),^7–10^ liquid chromatography-mass spectrometry (LC-MS),^11–14^ capillary electrophoresis (CE),^15–17^ electrochemical techniques,^18–20^ and fluorometric methods.^21–23^

Eosin Y (EY) is a fluorescent compound that has shown great potential as a sensing material for the determination of many drugs.^24,25^ This is due to the ability of those drugs to quench EY emission, which can be investigated using different mechanisms. Mechanisms of quenching involve either the interaction of the drug with EY through electron transfer,^26,27^ or more commonly through ion-pair complexation between the drug and EY.^28–30^ The use of EY as a sensing material has been demonstrated for the analysis of many drugs such as antibiotics,^31,32^ beta-blockers,^28^ and psychoactive drugs.^33^ The advantages of using EY for the drug determination include high sensitivity, simplicity, cost effectiveness, and low detection limits.^34^

While high performance liquid chromatography (HPLC) and mass spectrometry (MS) provide sensitive drug quantification, the high instrument costs and need for trained users limit accessibility. Fluorescent quantum dots and nanoparticles enable detection down to ng mL^−1^ levels, but require complex chemical synthesis and surface modifications. Electrochemical techniques are simple to perform but lack molecular selectivity. Immunoassays can be highly specific, though antibody development is labor-intensive. In contrast, EY is an inexpensive, commercially available fluorescent dye that enables direct drug detection down to ng mL^−1^ levels with a simple spectrofluorometer. The mix-and-measure approach using EY minimizes sample processing compared to extraction-intensive methods like HPLC. No probe fabrication steps are required, expanding accessibility to basic laboratory settings. EY provided recovery and accuracy equivalent to HPLC while being faster and simpler to perform. While previous reported methods utilizing EY for drug detection have demonstrated good sensitivity,^35–38^ our developed method offers even higher levels of sensitivity.

Carbon dots (CDs) are a promising type of nanomaterials due to their small size, remarkable biocompatibility, and remarkable fluorescence efficiency.^39^ Unlike other nanomaterials, CDs can be obtained from plant sources using safe and non-toxic synthetic approaches, making them safe for the use in various applications.^40^ Carbon dots offer unique advantages as fluorescent biosensing platforms compared to other nanoparticles like quantum dots or noble metal nanoclusters. The superior biocompatibility of carbon dots due to their non-toxic composition of carbon, oxygen and hydrogen permits their use in physiological environments with minimal perturbation.^40^ Additionally, the ease of surface functionalization through simple chemistry enables immobilization of various biomolecular recognition elements on carbon dots. Their excellent aqueous dispersibility facilitates bioconjugation and biosensing in solution. Furthermore, the excitation-dependent multicolor photoluminescence of carbon dots enables development of probes for more accurate detection.^40^ Carbon dots have been employed in biosensing applications, including the detection of biomolecules like glucose^41^ and DNA.^42^ Inspired by these findings, we used seedless black grapes (*Vitis vinifera*) as the precursor for synthesizing black grapes-derived carbon dots (BG@CDs) through a simple hydrothermal method. Black grapes (*Vitis vinifera*) were selected due to their rich skin composition of polyphenolic and other organic compounds, which facilitate efficient carbonization and yield a high photoluminescence quantum yield.^43,44^ This method is a popular technique for synthesizing CDs which involves the use of high pressure and high temperature to create the desired nanoparticles.^45^

A promising approach involves the use of two fluorescent materials that can be selectively quenched by KET and GNT has been developed. In this regard, the fluorometric sensor has been designed using EY and BG@CDs for the simultaneous determination of GNT and KET. The sensor exploits the differential quenching of fluorescence exhibited by the two fluorescent materials. Herein, GNT selectively quenches the fluorescence of EY through binary complex formation, while not affecting the fluorescence of BG@CDs. On the other hand, KET selectively quenches the fluorescence of BG@CDs, while not affecting the fluorescence of EY. This differential quenching response enables the selective and simultaneous determination of both analytes in a single measurement. The proposed fluorometric method utilizing EY and BG@CDs offer several advantages, comprising excellent sensitivity, low detection limits, and capability to quantify multiple analytes simultaneously.^46^ Additionally, the sensor is simple to prepare and cost-effective. The development of such a sensor has significant potential for applications in clinical monitoring of GNT levels in presence of KET, where the simultaneous detection of target analytes is required.

The proposed method was also employed to detect GNT in both spiked and real rabbit plasma samples following simultaneous administration of GNT and KET. In addition, a comprehensive evaluation of the fundamental pharmacokinetic parameters of GNT alone and GNT in the presence of KET is carried out. By using the fluorometric procedure and conducting a pharmacokinetic interaction investigation, we can gain a better understanding of how KET affects GNT level in the rabbit plasma samples. This information can be useful for developing effective treatment strategies and dosing regimens for patients receiving both drugs simultaneously. The sensitivity and selectivity of the method were greatly enhanced by the use of Oasis WCX cartridge as a mixed-mode solid-phase extraction (SPE) method, making the method an excellent tool for pharmacokinetic studies and therapeutic drug monitoring of GNT and KET.

In comparison to previously published work, our study provides a more rigorous analysis of the interaction between carbon dots and GNT. Although previous study has reported fluorescence quenching between carbon dots and GNT, and suggested the occurrence of a FRET mechanism, no single evidence was given to support their postulation.^47^ We believe that it is impossible for GNT to quench fluorescence of carbon dots due to its completely aliphatic structure, which makes the possibility of overlapping with the excitation spectrum very small. In addition, for FRET mechanism to occur, overlapping between absorption spectrum of the drug and emission spectrum of carbon dots is required, which is again impossible. Another paper also discusses quenching of carbon dots with GNT, but they report that GNT enhances quenching effect induced by lead ions, and no mechanism is discussed for this phenomenon.^48^ Overall, our study provides a more comprehensive analysis of the interaction between carbon dots and GNT, which enhances our understanding of this system.

## 2. Experimental

### 2.1. Materials and reagents

Ketorolac tromethamine (purity; 99.0%) was supplied by Amriya Pharmaceutical Industries (Alexandria, Egypt). Gentamicin sulfate (purity; 99.0%) was gifted by Memphis Pharmaceutical Industries (Cairo, Egypt). Eosin Y was purchased from Sigma-Aldrich (Steinheim, Germany). Quinine sulfate was purchased from Alfa Aesar (Ward Hill, MA). Ketolac® ampoules containing 30 mg KET per 2 mL and Garamycin® ampoules containing 80 mg GNT per 2 mL were obtained from local drug store. Britton–Robinson buffer with different pH values were prepared using phosphoric acid, boric acid, acetic acid with sodium hydroxide and those chemicals were obtained from El-Nasr Company for Intermediate Chemicals (Cairo, Egypt). All reagents were of analytical reagent grade.

### 2.2. Instrumentation

UV-visible absorption spectra were obtained using a Shimadzu UV-1601 spectrophotometer from Japan, equipped with a 1 cm quartz cell. Fluorescence emission spectra were measured using a Shimadzu RF-5301PC spectrofluorometer from Tokyo, Japan, with a slit width of 5 nm. Characterization of BG@CDs was also carried out using a Nicolet 6700 FT-IR spectrophotometer from Thermo Electron Corporation, USA, in the 400–4000 cm^−1^ range. The X-ray diffraction pattern for the BG@CDs was collected using a Philips PW 1700 X-ray diffractometer from Eindhoven, Netherlands. To estimate the morphology of the BG@CDs, a JEM-1400 Flash transmission electron microscope from JEOL in Tokyo, Japan was used. The mean particle sizes of the nanoparticles were determined using a ZetaPALS laser particle size analyzer from Brookhaven. X-ray photoelectron spectrometer (XPS, ESCA Ulvac-PHI 1600, PHI Quantum 2000 XPS system, Physical Electronics, USA) was used to reveal the surface functional groups of BG@CDs.

### 2.3. Preparation of BG@CDs

The BG@CDs were prepared through hydrothermal treatment of black grape juice. Fresh seedless black grapes (200 g) were washed with double distilled water and blended to extract 100 mL of grape juice. The juice was transferred to a 200 mL Teflon-lined stainless steel autoclave reactor (filling factor of 50%). Hydrothermal carbonization was carried out at 180 °C for 10 hours. After cooling, the product was centrifuged at 5000 rpm for 10 minutes to isolate the dots. The filtrate containing BG@CDs was passed through a 0.22 μm syringe filter to remove larger particles. The final BG@CDs were lyophilized, giving a yield of approximately 40 mg and the obtained product was collected for future use.

### 2.4. Simultaneous measurement of GNT and KET

The fluorescent sensor solution was prepared using two fluorescent materials: eosin Y (1.0 μg mL^−1^) and BG@CDs (10.0 μg mL^−1^). Firstly, 2.0 mL BR buffer solution (pH 5.0), 1 mL EY and 1 mL BG@CDs were placed into 10 mL calibrated volumetric flasks. Then, different volumes of stock solutions of GNT (10.0 μg mL^−1^) and/or KET (10.0 μg mL^−1^) were added. Secondly, DDW was added to complete the volume to 10 mL to obtain the final concentrations ranging from 50.0 to 500 ng mL^−1^ for GNT and from 20.0 to 400 ng mL^−1^ for KET. Two emission peaks were observed after excitation at 310 nm: one at 385 nm for BG@CDs and the other at 535 nm for EY. When GNT was added to the sensor, it resulted in the quenching of the EY peak at 535 nm. On the other hand, when KET was added to the sensor, it led to the quenching of the BG@CDs peak at 385 nm. The fluorescence emission was measured within a reaction time of one minute.

### 2.5. Animals

This testing received full ethical approval from the ethics committee at Assiut university, Egypt (05-2023-006) and was conducted in concordance to declaration of Helsinki principals. The study involved six New Zealand white rabbits, each weighing 2.0 ± 0.2 kg, that were purchased from a licensed animal supplier in Assiut, Egypt. Before the study began, the rabbits were assessed to be in good health. They were housed in individual cages that measured 0.8 × 0.6 × 0.5 m and given 15 days to acclimate to the experimental conditions and handling, during that time, they were not given any medications or vaccines. The room temperature was held constant at 25 ± 2 °C and the relative humidity was kept at 50 ± 5%. Throughout the acclimatization period, the rabbits were provided with a commercial pellet diet and water. Prior to the start of the experiment, the rabbits fasted for almost 12 h, but had access to water.

### 2.6. Preparation of calibration standards

Seven calibration samples were produced by adding suitable quantities of the stock solution of GNT (10.0 μg mL^−1^) and KET (10.0 μg mL^−1^) to drug-free rabbit plasma. The final concentrations of the calibration samples after extraction and dilution were from 60 to 500 ng mL^−1^ for GNT and from 25 to 400 ng mL^−1^ for KET. The spiked plasma samples were collected and stored in tightly sealed, dark containers at −20 °C. The plasma samples were allowed to thaw at room temperature before analysis.

### 2.7. Sample preparation and extraction procedures

To extract GNT and KET from spiked rabbit plasma samples, acetonitrile was used as a protein precipitating agent. This involved adding 3 mL of acetonitrile to 1.0 mL of each spiked plasma sample in a centrifuge tube. The tubes were then vortex mixed for 2 min and centrifuged at 8000 rpm for 10 min. After centrifugation, the supernatant was transferred to clean test tube. After protein precipitation the supernatant was further purified with reported solid phase extraction (SPE) clean-up procedures with polymeric weak cation exchange (WCX) cartridges specifically designed for aminoglycosides.^49,50^ To prepare the sample for solid phase extraction (SPE) using the Oasis WCX cartridge, the pH of the supernatant was first adjusted to 7.0 by adding few drops of 2.0 M NaOH solution. The cartridge was then conditioned with 4 mL of methanol and 4 mL of DDW before the sample was loaded onto it. Once the sample was loaded, the cartridge was rinsed with 6 mL of DDW and dried under vacuum for 2 min. After drying, the analytes were eluted from the cartridge using 5 mL of 40% formic acid in methanol. The eluate was then vortexed and filtered into polypropylene vials before being diluted with DDW for fluorometric analysis. Ultimately, this method yielded final concentrations of 60–500 ng mL^−1^ for GNT and 25–400 ng mL^−1^ for KET.

### 2.8. Pharmacokinetic application

Six rabbits were used in this study. The rabbits administered GNT *via* intravenous route at a dose of 4 mg kg^−1^. After a washout period of 21 days to allow any GNT to clear from the rabbits' bodies, the same rabbits administered both (4 mg kg^−1^ for GNT and 1.5 mg kg^−1^ for KET). Blood samples (1 mL) were collected after administration of the studied analytes after 0.25, 0.5, 1, 1.5, 3, 5, 7, 5, 10, 14, 18 and 24 h of their administration. The samples were drawn from an ear vein, placed into tubes that had been heparinized, and centrifuged for 15 minutes at 6000 rpm. The plasma was then collected and stored at −20 °C in dark containers until analysis. The pharmacokinetic parameters of GNT were calculated using two compartment model for the two treatment groups using Phoenix®WinNonlin software version 5.1 (Pharsight, Mountain View, CA, USA). These parameters included total area under the curve (AUC) from 0 to 24 hours (AUC_0→24_), cumulative plasma concentration from 0 to infinity (AUC_0→∞_), peak plasma concentration (*C*_max_), elimination rate constant (*K*_el_), distribution half-life (*t*_1/2_α), elimination half-life (*t*_1/2_β), volume of distribution in the central compartment (*V*_c_), volume of distribution in the peripheral compartment (*V*_p_), clearance (CL). The data are reported as mean ± standard deviation (mean ± SD). To analyze differences in parameters between the groups, *t*-test was employed, using GraphPad Prism 9.0. A *p*-value < 0.05 was found to be statistically significant, indicating differences between paired data sets.

## 3. Results and discussion

### 3.1. Characterization of BG@CDs

**Fig. 1:**
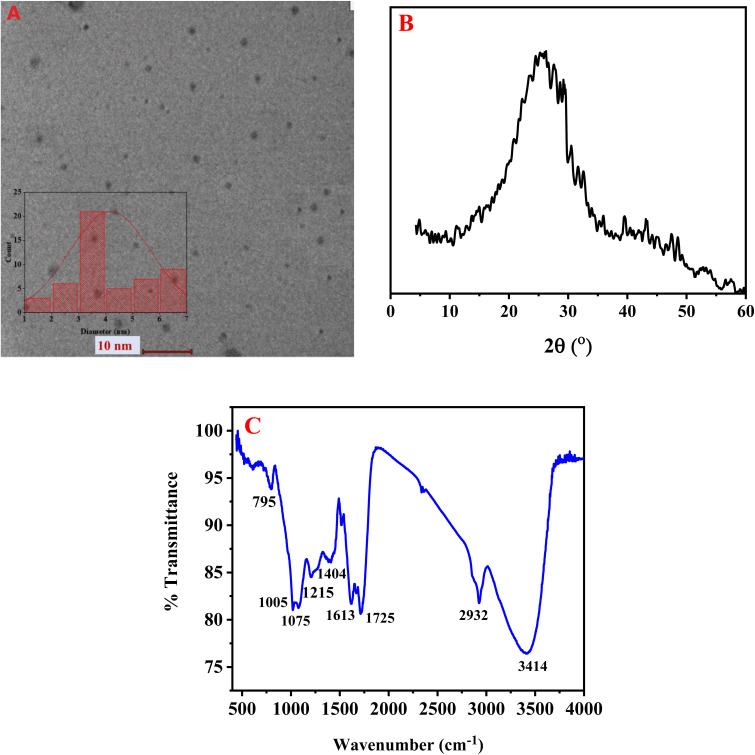
(A) TEM images, (B) XRD pattern, (C) FT-IR of BG@CDs.

Fig. 1A displays the transmission electron microscopy (TEM) image of the synthesized BG@CDs, revealing mostly uniform spherical particles with good dispersion. The particle size frequency distribution histogram indicates that the BG@CDs with most abundant size ranging from 3 to 4 nm (inset). To assess the BG@CDs crystalline structure, X-ray diffraction (XRD) was employed. As depicted in Fig. 1B, the XRD pattern displays a relatively broad diffraction peak at 2*θ* = 25.82°, corresponding to the (0 0 2) diffraction plane of partially disordered graphitic structures, indicating the amorphous nature of the BG@CDs. Fig. 1C presents the findings from FTIR analysis of the synthesized BG@CDs. The stretching and bending vibrations of different molecular bonds were held responsible for the absorption bands that were detected in the spectrum. Specifically, the peak at 3414 cm^−1^ was attributed to the stretching vibration of O–H/N–H bonds.^51^ The peak at 2932 cm^−1^ was associated with the C–H stretching modes.^52^ The absorption bands at 1725 cm^−1^ and 1613 cm^−1^ corresponded to the stretching vibration of the C=O/C=N and bending vibration of NH/OH, respectively.^53^ A peak at 1404 cm^−1^ corresponds to C–NH–C groups, and peaks at 1215 cm^−1^, 1075 cm^−1^ and 1005 cm^−1^ were assigned to vibration bands of C–C, C–N, C–O, respectively.^54^ Finally, the band observed at 795 cm^−1^ corresponded to the N–H bending (out-of-plane) vibrations. The existence of strong absorption bands at 3414 cm^−1^ indicated that the surface of the BG@CDs contained abundant amine and carboxylic groups, which contributed to their hydrophilicity.^55^ Fig. S1A† exhibits the XPS analysis of BG@CDs with prominent peaks at 285.4 eV, 396.6 eV, and 528.7 eV, corresponding to C 1s, N 1s, and O 1s, respectively. Fig. S1B† represents C 1s spectrum with sharp peaks at 282.2 eV, 283.2 eV, and 284.5 eV, which assign to C=C/C–C, C–N, and C=O/C=N, respectively.^56^ Fig. S1C† represents N 1s spectrum with prominent peaks at 397.2 eV and 398.3 eV, assigning to pyridinic N and amidic/amino N, respectively.^55^ Fig. S1D† shows the O 1s spectrum with distinctive peaks at 528.6 eV and 529.2 eV, corresponding to C=O and C–OH/C–O–C, respectively.^57^

### 3.2. Optical properties and stability of the prepared BG@CDs

**Fig. 2:**
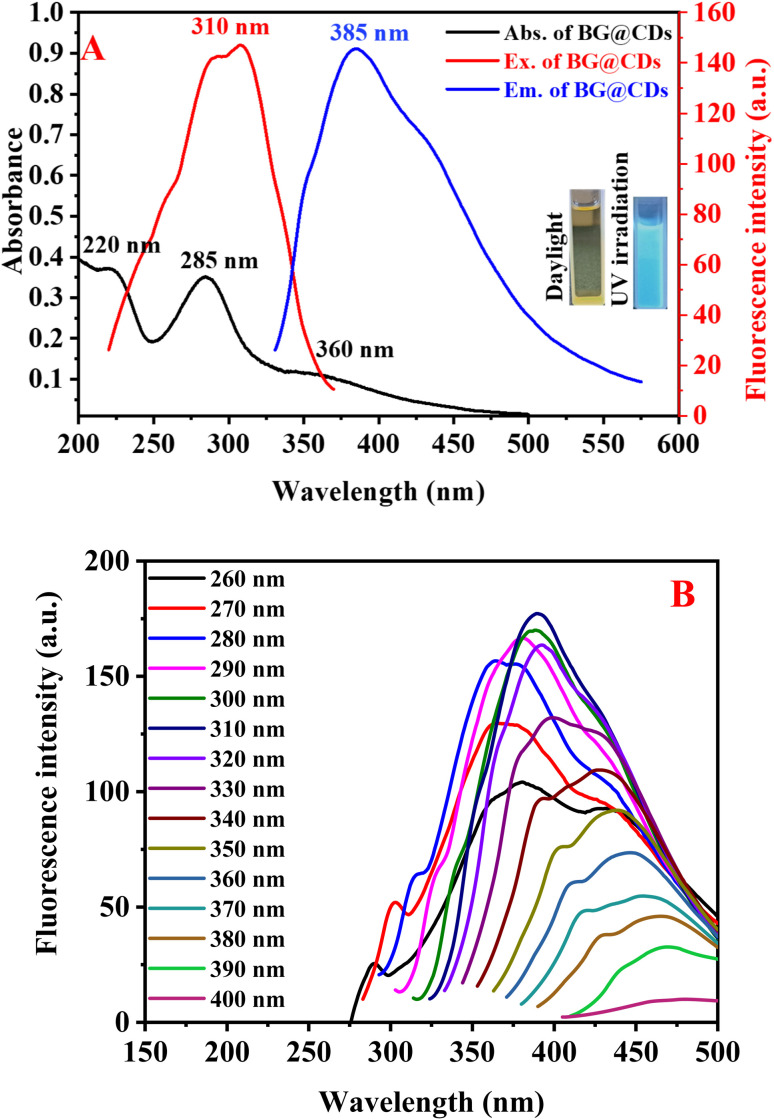
(A) Overlay of UV-vis absorption spectra, excitation spectra and emission spectra of BG@CDs (0.1 mg mL^−1^). Inset: the images of BG@CDs under visible light and UV irradiation at 365 nm. (B) The fluorescence emission spectra of BG@CDs (0.1 mg mL^−1^) at different excitation wavelengths (from 260 to 400 nm).

The optical behavior of as-synthesized BG@CDs were investigated by measuring UV-vis and fluorescence spectra. An intense peak at 285 nm and shoulder peak at 220 nm in the UV absorption spectrum of the BG@CDs are assigned to the π–π* transition of the C=C bonds, and a broad peak at 360 nm is caused by the n–π* transition of the C=O bonds on the BG@CDs (Fig. 2A). The fluorescence spectra of BG@CDs show that the optimal excitation and emission wavelengths are 310 nm and 385 nm, respectively (Fig. 2A). The emission spectra of the BG@CDs in the aqueous solution recorded at different excitation wavelengths (Fig. 2B). The emission peak shows excitation-dependent emission pattern when the excitation wavelength varied from 260 to 400 nm. These features are characteristic of the quantum confinement luminescence due to different size and surface states of BG@CDs.^58^ The strongest emission peak was observed under 310 nm excitation. The prepared BG@CDs displayed a bright-blue fluorescence upon UV irradiation (Fig. 2A inset). Furtherly, the quantum yield of the prepared BG@CDs was determined to be 23% by using quinine sulfate (QY = 0.54) as reference, confirming that the BG@CDs have reasonable fluorescence emission. The measurement of fluorescence quantum yield of BG@CDs was described in details at ESI.†

The stability investigation focused on the effect of various factors on the fluorescence intensity of BG@CDs, including pH variation, ionic strength, and the length of UV irradiation. Fig. S2A† demonstrates the influence of different pH levels (1–11) on fluorescence intensity. Upon changing the pH, the acidic medium with a pH below 5 exhibited a stronger suppression of fluorescence intensity, while the intensity experienced only slight variations within the pH range of 5–11. This phenomenon may be related to the protonation or deprotonation of functional groups.^59,60^ The impact of ionic strength on the fluorescence properties of BG@CDs was examined and depicted in Fig. S2B.† The fluorescent emission of BG@CDs remained stable even when the concentration of NaCl was elevated to 1.0 M. Moreover, as illustrated in Fig. S2C,† BG@CDs demonstrated impressive durability against photo-bleaching when exposed to constant UV light for 240 minutes with a wavelength of 365 nm.

The influence of various patches obtained from different suppliers of black grapes (*Vitis vinifera*) on the fluorescence intensity of BG@CDs was examined. Notably, the fluorescence intensity was shown in Fig. S2D,† which revealed no significant variation in the fluorescence intensity across five different suppliers. This observation suggests that the fluorescence intensity of carbon dots obtained from black grapes remains consistent irrespective of the supplier, highlighting the robustness and reliability of the BG@CDs synthesis process.

### 3.3. Experimental parameters optimization

An extensive investigation was carried out to explore and enhance all the factors influencing the formation of the binary complex between GNT and EY. This comprehensive analysis included several variables, including the pH, the volume of buffer, the type of diluting solvent, as well as the volume of EY. The most critical variable in the formation of ion pair complex is the pH value of the solution as the ionization of GNT and EY is influenced by pH levels. Multiple pH values were investigated using BR buffer over pH range of 3.0–9.0 (Fig. S3A†). It was found that, the quenching effect increased as pH values elevated up to pH 5.0 and slight change was noticed at pH above 7. GNT possesses five nitrogen atoms with basic properties, which exhibit a p*K*_a_ range of 5.7–10.0.^61^ Therefore, a pH level of 5 was deemed suitable for the complete ionization of all the nitrogen atoms in GNT, causing it to carry a positive charge. Also, EY as acidic dye will be in di-ionic form when pH is higher than 4.9 as eosin highest p*K*_a_ value is 4.9.^62^ So, pH 5 assures strongest electrostatic attraction of EY with GNT. Various volumes of BR buffer varied from 0.5 mL to 3.0 mL were tested and it was found that the highest fluorescence quenching was obtained with a volume of 2.0 mL as shown in Fig. S3B.† As a result, 2.0 mL of BR buffer with a pH of 5 were used for the rest of the study. Concentration of EY (1.0 μg mL^−1^) was sufficient to produce a high intensity peak sufficient for quenching after adding GNT. Different EY volumes were tested in range from (0.1–2.0 mL), it was noted that 1 mL of EY (1.0 μg mL^−1^) was the optimal volume as it was enough to give the highest Δ*F* (Fig. S3C†). Several diluting solvents, including distilled water, acetonitrile, methanol, and ethanol were evaluated to determine the optimal solvent for achieving the highest response from the developed sensor (Fig. S3D†). Among these solvents, distilled water was found to be the most effective in producing the highest Δ*F* values, surpassing the performance of the other solvents.

The quenching effect of KET on BG@CDs fluorescence was examined using BR buffer at different pH levels ranging from 5.0 to 11.0. Interestingly, no significant difference in the fluorescence intensity of KET was detected across the studied pH range (Fig. S4A†). Ketorolac tromethamine is a salt that is formed by combining ketorolac, which is an acidic compound, with tromethamine, which is a basic compound. The combination of these two compounds results in a neutral and water-soluble salt. So, it is possible that the formed salt may be independent of the pH of the solution which explain this behavior and pH 5 was chosen for subsequent measurement as it gives good response for the simultaneous analysis. Additionally, the optimal BG@CDs volume for quenching the fluorescence of BG@CDs was determined to be 1.0 mL, following examination of volumes from 0.5 mL to 3.0 mL (Fig. S4B†). After investigating a concentration range from 0.01 mg mL^−1^ to 1.0 mg mL^−1^ (Fig. S4C†), it was concluded that the ideal concentration of BG@CDs is 0.1 mg mL^−1^. The impact of reaction time was also investigated, and it was discovered that the entire reaction took less than a minute, as evidenced by the measured fluorescence emission taken at intervals of one to ten min. (Fig. S4D†). Additionally, the fluorescence signal remained stable over three hours.

### 3.4. Simultaneous sensing of GNT and KET

**Fig. 3:**
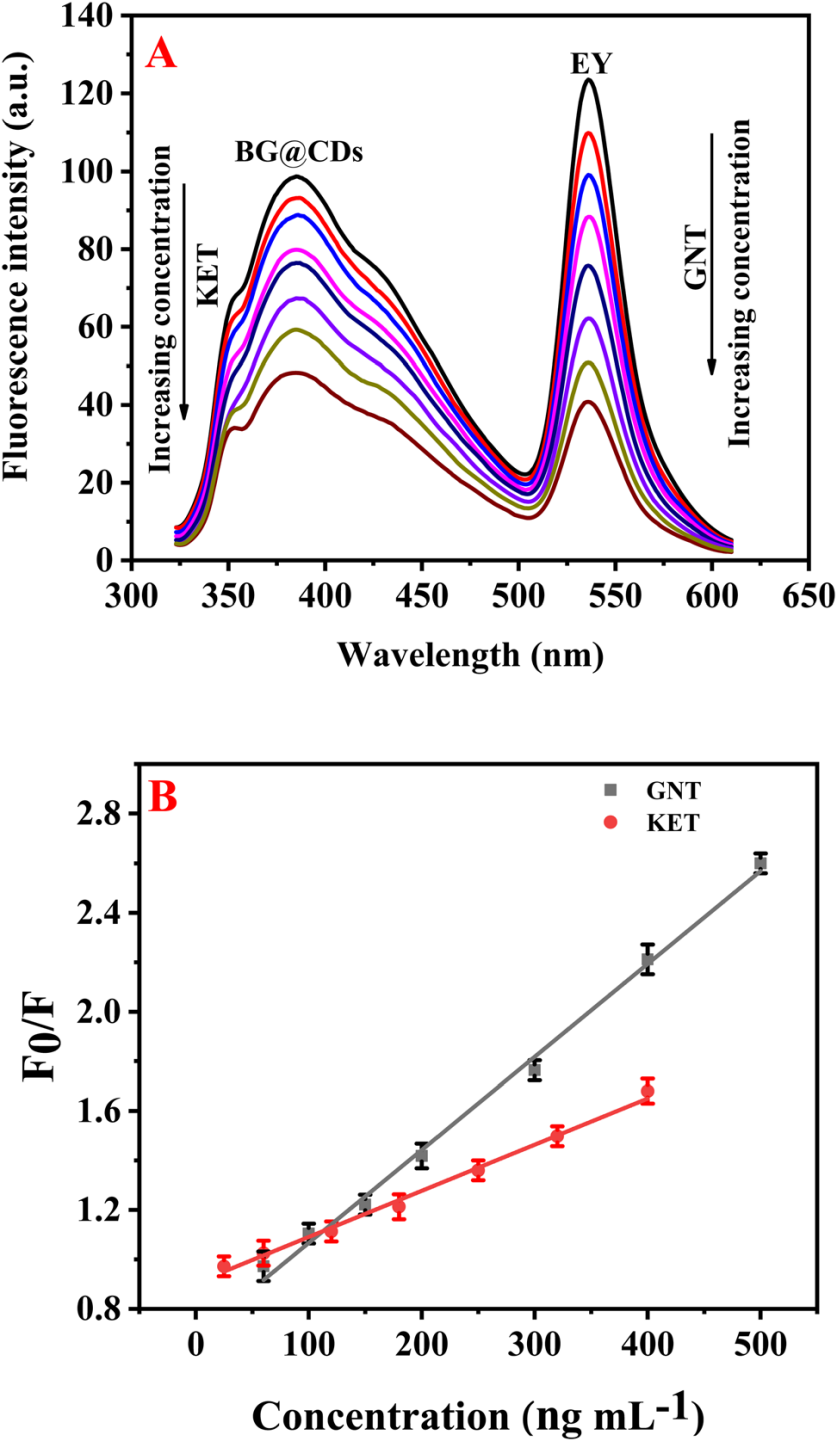
(A) The influence of different concentrations of GNT (50–500 ng mL^−1^) and KET (20–400 ng mL^−1^) on the fluorescence emission of EY and BG@CDs, respectively. (B) Plot of (*F*_0_/*F*) *versus* concentration of GNT/KET.

Upon optimizing the experimental parameters for the detection of GNT and KET, it was found that these drugs can be simultaneously determined using EY/BG@CDs as a fluorescent sensor. In this system, KET was observed to quench the fluorescence of BG@CDs while not affecting the fluorescence of EY (Fig. S5A†). In contrast, GNT was found to quench the fluorescence of EY but did not impact the fluorescent signal of BG@CDs (Fig. S5B†). This unique combination of properties enables the detection of both analytes using a single sensor system, providing an easy and reliable approach for the simultaneous analysis of GNT and KET (Fig. 3A). The calibration curves of GNT and KET were constructed by plotting concentration *versus F*_0_/*F* of the prepared EY/BG@CDs sensor. The constructed calibration curves showed good linearity across the range of concentrations from (50–500 ng mL^−1^ for GNT and 20–400 ng mL^−1^ for KET) as shown in Fig. 3B. The obtained regression equations can be represented as the following:*F*_0_/*F* = 0.0037*C*_GNT_ + 0.69 (*R*^2^ = 0.9983)*F*_0_/*F* = 0.0018*C*_KET_ + 0.91 (*R*^2^ = 0.9982)where *F*_0_ and *F* are the fluorescence intensities of sensor with and without the target analytes. The limits of detection (LOD) for GNT and KET were found to be 15.68 and 6.44 ng mL^−1^, respectively, whereas the limits of quantification (LOQ) were calculated to be 47.50 and 19.51 ng mL^−1^, respectively.

### 3.5. Sensing mechanism

**Fig. 4:**
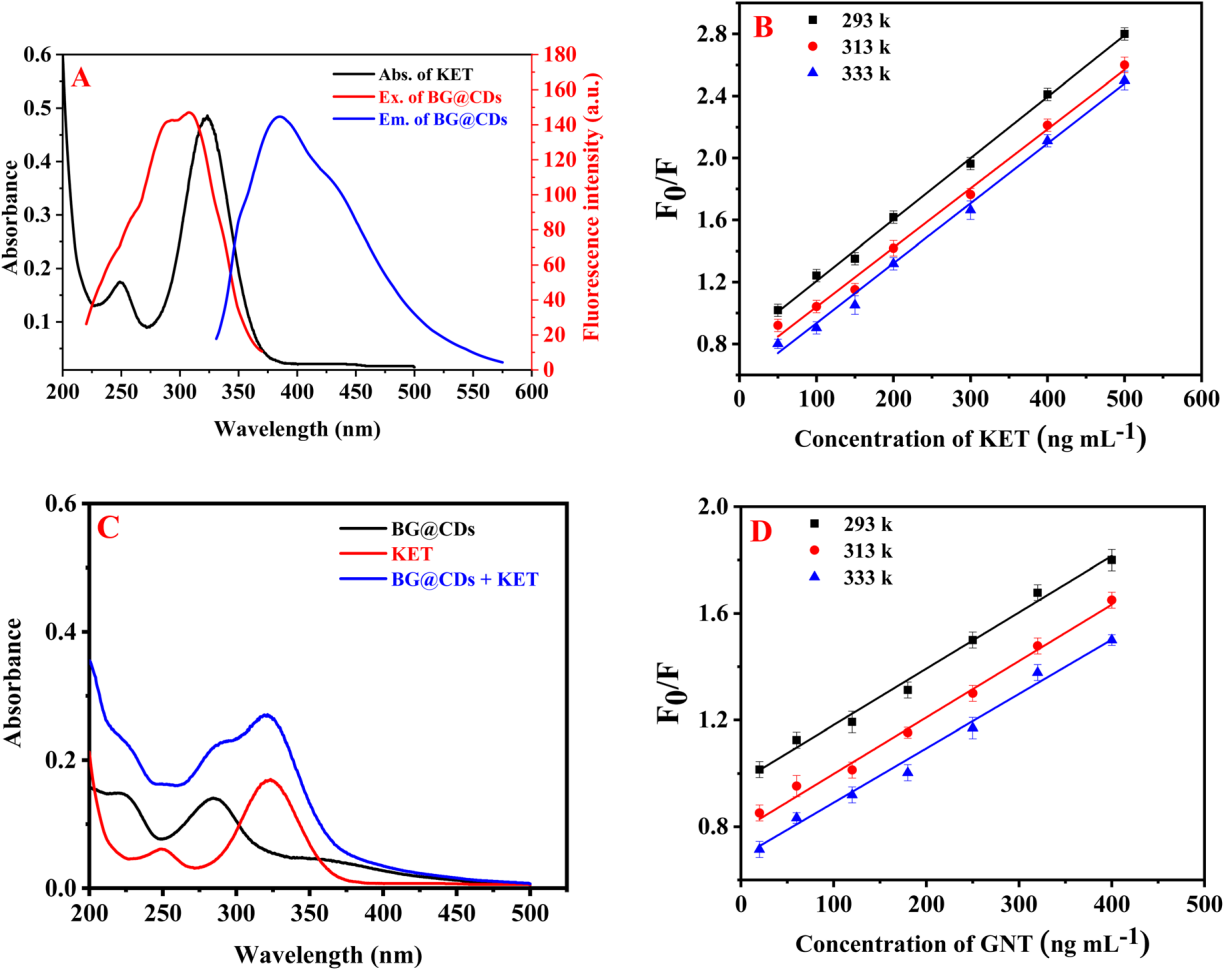
(A) UV-vis absorption of KET (10.0 μg mL^−1^) and excitation/emission spectra of the BG@CDs (0.1 mg mL^−1^), (B) Stern–Volmer plots of BG@CDs (0.1 mg mL^−1^) with KET under different temperatures, (C) UV-vis absorption spectra of BG@CDs (0.1 mg mL^−1^), KET (5.0 μg mL^−1^) and their mixtures, (D) Stern–Volmer plots of EY (0.1 μg mL^−1^) with GNT under different temperatures.

The results revealed that the use of KET effectively quenches the fluorescence of BG@CDs. Fig. 4A shows significant overlap between the UV spectrum of KET and the excitation spectrum of BG@CDs, indicating that fluorescence quenching is related to inner-filter effect (IFE).^54^ To calculate the fluorescence quenching efficiency (*F*_0_/*F*) of BG@CDs in the presence of KET, the Stern–Volmer equation was used: *F*_0_/*F* = *K*_sv_[Q] + 1.^63,64^ Here, *F*_0_ and *F* represent the fluorescence of BG@CDs in the absence and presence of KET, respectively. *K*_sv_ denotes the Stern–Volmer quenching constant, and [Q] express the concentration of KET solution. When it comes to static quenching, the *K*_sv_ decreases as the temperature increases. However, in the case of dynamic quenching, the *K*_sv_ increases with increasing temperature.^65^ A Stern–Volmer plot was used to examine the fluorescence quenching between *F*_0_/*F* and [Q] at three different temperatures (293 K, 313 K, and 333 K) as shown in Fig. 4B. Each KET concentration curve was found to be linear, and with increasing temperature, the slope (*K*_sv_) of each curve decreased. This indicates that the quenching process observed in the study was a static quenching process. To further investigate the quenching mechanism, UV-vis spectra of BG@CDs, KET, and BG@CDs with KET were recorded. As illustrated in Fig. 4C, the summed spectra indicated a slight shift in wavelength occur when BG@CDs were added to KET. The addition of KET caused absorption changes in the system, which could indicate the complex formation in ground state between BG@CDs and KET.^66^ Taken together, the sensing behavior of BG@CDs with KET satisfies almost all criteria of static quenching mechanism.^67^ The results suggest that the combined influence of IFE and static quenching is responsible for the observed fluorescence quenching.

The quenching of EY native fluorescence was found to occur not only through ion–pair complex formation, but also through dynamic quenching,^68^ static quenching,^69^ or fluorescence resonance energy transfer. To investigate the fluorescence quenching mechanism between EY and GNT, the Stern–Volmer equation was utilized. Results showed that the *K*_sv_ decreased with elevating temperature as shown in Fig. 4D, indicating that the process of quenching is of a static nature.

### 3.6. Selectivity of the proposed sensor

To confirm the selectivity of the fluorometric sensor for detecting GNT and KET in rabbit plasma, several antibiotics and NSAIDs were chosen in this study, including neomycin, erythromycin, vancomycin, ibuprofen, naproxen, and diclofenac. We also investigated the interference of various possible substances in plasma, such as proteins and amino acids (*e.g.*, bovine serum albumin, l-arginine, cysteine, glycine, and proline) and metal ions (*e.g.*, Ca^2+^, Mg^2+^, Na^+^, Fe^2+^, K^+^, Fe^3+^, Cu^2+^, Zn^2+^, Co^2+^ and Ni^2+^). As shown in Fig. S6A and S6B,† the different kinds of interfering species had almost no effect on the fluorescence intensity of either BG@CDs or EY. These results prove that the sensor is selective for detecting GEN and KET.

### 3.7. Extraction efficiency

After extraction using protein precipitation with acetonitrile, plasma was further purified using mixed mode solid phase extraction cartridge. The Oasis WCX cartridge is a suitable choice for separating GNT and KET from plasma matrix due to its unique properties. GNT is a positively charged, polar molecule, while KET is a neutral, polar molecule. The weak cation exchange (WCX) groups in the stationary phase of the Oasis WCX cartridge can selectively bind to positively charged molecules, such as GNT. At the same time, the hydrophilic surface of the cartridge can effectively retain polar molecules, such as KET. Therefore, the Oasis WCX cartridge can efficiently extract and separate both compounds from plasma matrix, providing high recoveries and purity for subsequent analysis. Additionally, the high surface area of the hydrophilic polymer in the Oasis WCX cartridge ensures efficient sample extraction and minimizes matrix effects. Table 1 presents a summary of the extraction recoveries and matrix effect obtained from the results. The mean extraction recoveries ranged from 98.05 to 98.62% for GNT, and from 97.02.4 to 98.23% for KET. All the results indicate that the extraction recovery and matrix effect were consistent and reproducible.

**Table 1:** Analyzing plasma samples at three different concentrations utilizing a recommended technique and the Oasis WCX cartridge as a sorbent material (*n* = 3)

Drug	Added (ng mL^−1^)	Found (ng mL^−1^)	Recovery (%) ± RSD (%)
GNT	100.0	98.05	98.05 ± 1.06
	200.0	197.23	98.62 ± 1.41
	400.0	392.56	98.14 ± 1.35
KET	60.0	58.21	97.02 ± 2.14
	180.0	177.2	98.44 ± 1.81
	320.0	314.32	98.23 ± 1.79

### 3.8. Method applications

#### 3.8.1. Sensing of KET and GNT in spiked rabbit plasma samples

The proposed method aims to assess the suitability of a developed procedures for quantifying the concentrations of the studied drugs in plasma samples. When GNT or KET are administered intravenously at their therapeutic levels, their reported plasma concentration is between (1000.0–20 000 ng mL^−1^ for GNT and 2000.0–20 000 ng mL^−1^ for KET).^5,70^ The proposed method was effectively utilized to measure the concentrations of both GNT and KET in plasma samples obtained from rabbits and the found determination range was (60.0–500 ng mL^−1^ for GNT and 25.0–400 ng mL^−1^ for KET) (Fig. S7A and B†). The regression equations that correspond to the obtained results are as follows:*F*_0_/*F* = 0.004*C*_GNT_ + 0.81 (*R*^2^ = 0.9976)*F*_0_/*F* = 0.002*C*_KET_ + 0.97 (*R*^2^ = 0.9916)

The limits of detection (LOD) for GNT and KET were estimated to be 18.97 and 7.92 ng mL^−1^, respectively, whereas the limits of quantification (LOQ) were calculated to be 57.50 and 24.01 ng mL^−1^, respectively.

#### 3.8.2. Pharmacokinetic application

**Fig. 5:**
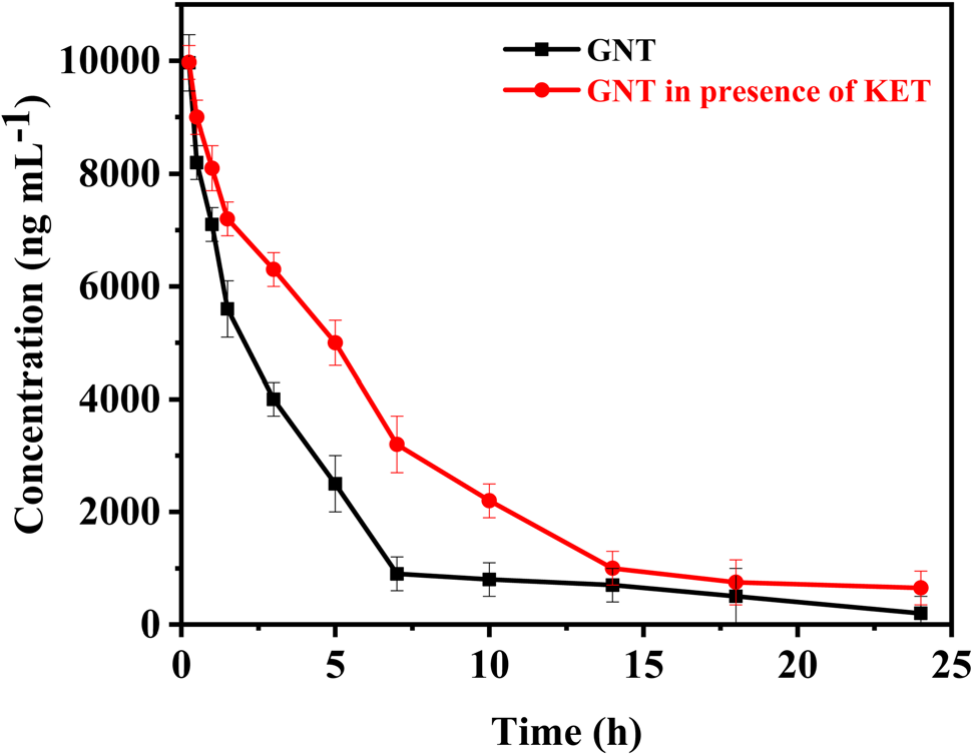
The mean concentrations of GNT *vs.* time profile, in absence and after administration of single I.V. dose of KET.

The proposed spectrofluorometric method was successfully applied for the quantification of GNT in presence of KET in plasma samples for application to pharmacokinetic study in rabbits. Calibration ranges were found suitable to detect samples obtained after co-administration of GNT (1.5 mg kg^−1^) and KET (4.0 mg kg^−1^) in rabbits. The highest and lowest plasma levels in samples collected for pharmacokinetic evaluation were found within the calibration range of both drugs. Fig. 5 shows the pharmacokinetic profile of GNT in presence of KET. The computed pharmacokinetic parameters are summarized under Table 2.

**Table 2:** Comparison of the pharmacokinetics of GNT alone and in combination with KET following intravenous administration in rabbits (*n* = 3)

Parameters	GNT	GNT in presence of KET
*C*_max_ (ng mL^−1^)	9963.8 ± 781.07	9972.2 ± 645.12
*t*_1/2_α (h)	0.5 ± 0.074	0.7 ± 0.058^a^
*t*_1/2_β (h)	1.4 ± 0.87	1.7 ± 0.18^a^
*V*_c_ (L kg^−1^)	0.25 ± 0.042	0.3 ± 0.089^a^
*V*_p_ (L kg^−1^)	0.15 ± 0.023	0.1 ± 0.045^a^
*k*_el_ (h^−1^)	0.69 ± 0.048	0.59 ± 0.073^a^
CL (L kg^−1^ h^−1^)	2.5 ± 0.51	2.2 ± 0.71^a^
MRT (h)	1.8 ± 0.23	2.0 ± 0.85^a^
AUC_0–24_ (ng hr mL^−1^)	134 500 ± 7955.56	142 000 ± 8647.02^a^
AUC_0–∞_ (ng hr mL^−1^)	269 000 ± 9850.81	284 000 ± 9735.45^a^

a Indicates statistically significant differences between parameters values of GNT and GNT/KET (*p* < 0.05).

When KET is administered with GNT, several changes in pharmacokinetic parameters may occur. The distribution and elimination half-lives of GNT are longer in this case compared to when GNT is administered alone. This prolonged half-life may be attributed to the effect of KET on the volume of distribution of GNT, which could decrease the rate of distribution into the peripheral compartment. Moreover, administering KET with GNT can also lower the clearance of GNT due to ketorolac's impact on renal function. It is known that nonsteroidal anti-inflammatory drugs (NSAIDs) can decrease renal blood flow and glomerular filtration rate (GFR), which in turn reduces the clearance of renally eliminated drugs like GNT. Furthermore, the AUC of GNT is higher when administered with KET, this increase in AUC may be due to the lower clearance and longer distribution half-life of GNT in this case. The higher AUC can result in higher concentrations of GNT over a longer period of time, which may increase the efficacy and/or toxicity of the drug. Lastly, the mean residence time of GNT is longer when administered with KET. This is consistent with the longer distribution half-life and lower clearance observed in this case. Overall, the changes in pharmacokinetic parameters after co-administration of KET with GNT are relatively modest. These findings suggest that KET pre-treatment can affect normal kidney function, which is the primary organ responsible for GNT excretion. The change in kidney function caused by KET can result in GNT accumulation at toxic levels, leading to further nephrotoxicity, ototoxicity, and neuromuscular paralysis. The increase in GNT levels is believed to be due to the reduced glomerular filtration rate. Therefore, it is suggested that the dosage of GNT should be reduced before starting KET therapy. To adjust the GNT dose, it is necessary to carefully monitor its plasma concentrations and evaluate KET-induced changes in renal function. These observations highlight the importance of conducting clinical studies to validate these findings.

## 4. Conclusion

In conclusion, this study presents a novel and highly sensitive method for the simultaneous determination of gentamicin sulphate (GNT) and ketorolac tromethamine (KET) in rabbit plasma samples using a sensor based on eosin Y and black grapes derived carbon dots (EY/BG@CDs). The BG@CDs were derived from black grapes, making the method environmentally friendly. GNT was found to quench the fluorescence of EY, while not affecting the fluorescence of BG@CDs. In contrast, KET quenched the fluorescence of BG@CDs, but did not affect EY fluorescence. The proposed method is simple, green, and sensitive, making it an attractive alternative to traditional chromatographic methods. Furthermore, the use of Oasis WCX cartridge as an efficient extraction method ensured the accuracy and reliability of the results obtained using the developed sensor. The analysis of the of both GNT and KET is of great importance due to the potential pharmacokinetic interactions between them. The study revealed that KET pre-treatment can affect normal kidney function, which is the primary organ for GNT excretion. The increase in GNT levels is believed to be due to the reduced glomerular filtration rate. Therefore, it is strongly recommended that the dosage of GNT should be reduced before starting KET therapy. Overall, this study provides a significant advancement in the development of sensitive and reliable methods for simultaneous drug analysis, with important applications in clinical pharmacology and toxicology fields. The findings can also be used for better therapeutic drug monitoring and personalized medicine. The developed method can be extended to the analysis of other drugs with similar properties, broadening its applicability.

## Conflicts of interest

The authors declare that they have no known competing financial interests or personal relationships that could have appeared to influence the work reported in this paper.

## Supplementary Material

RA-013-D3RA04894B-s001
