# Supplementary material for: A selective dual quenching sensor (EY/BG@CDs) for simultaneous monitoring of gentamicin and ketorolac levels in plasma: a highly efficient platform that caters to the needs of therapeutic drug monitoring
Source: RSC Adv. 2023 Oct 3;13(41):28940–50. doi: 10.1039/d3ra04894b (PMC10545982; doi:10.1039/d3ra04894b)
Supplement: RA-013-D3RA04894B-s001 [file RA-013-D3RA04894B-s001.pdf]

Electronic Supplementary Material (ESI) for RSC Advances.
This journal is © The Royal Society of Chemistry 2023

Electronic Supplementary Material (ESM)

"A Selective Dual Quenching Sensor (EY/BG@CDs) for Simultaneous Monitoring of Gentamicin and Ketorolac Levels in Plasma: A highly efficient platform that caters to the needs of therapeutic drug monitoring"

Khalid Alhazzani[a], Ahmed Z. Alanazi[a], Aya M. Mostafa[b,c], James Barker[b], Mohamed M. El-Wekil[c], Al-Montaser Bellah H. Ali[c*]

[a] Department of Pharmacology and Toxicology, College of Pharmacy, King Saud University, Riyadh, Saudi Arabia

[b] School of Life Sciences, Pharmacy, and Chemistry, Kingston University, Kingston-upon-Thames, London KT1 2EE, UK.

[c] Department of Pharmaceutical Analytical Chemistry, Faculty of Pharmacy, Assiut University, Assiut, Egypt.

*e-mail: Almontaser_bellah@aun.edu.eg

Fluorescence quantum yield of BG@CDs

The quantum yield (QY) of BG@CDs was calculated according to the following equation using quinine sulfate (QS) as a reference in 0.1 mol/L H_2SO_4 (QY = 54 %). By measuring the absorbance (less than 0.05) and emission spectra of a certain concentration of BG@CDs and quinine sulfate at the same excitation wavelength at 360 nm, the absorbance and fluorescence integral area were substituted into the following formula:

$$\phi_{BG@CDs} = \phi_{QS} \times \frac{F_{BG@CDs}}{F_{QS}} \times \frac{A_{QS}}{A_{BG@CDs}} \times \frac{\eta_{BG@CDs}}{\eta_{QS}}$$

$\phi_{BG@CDs}$ represents the quantum yield of BG@CDs, ϕ_{QS} represents the quantum yield of QS, $F_{BG@CDs}$ is the fluorescence intensity of BG@CDs, F_{QS} is the fluorescence intensity of quinine sulphate, A refers to the absorbance value and η refers to the refractive index of the solvent (distilled water). The synthesized BG@CDs were dissolved in distilled water (η = 1.33) and quinine sulfate was dissolved in 0.1 M H_2SO_4 (η = 1.33).

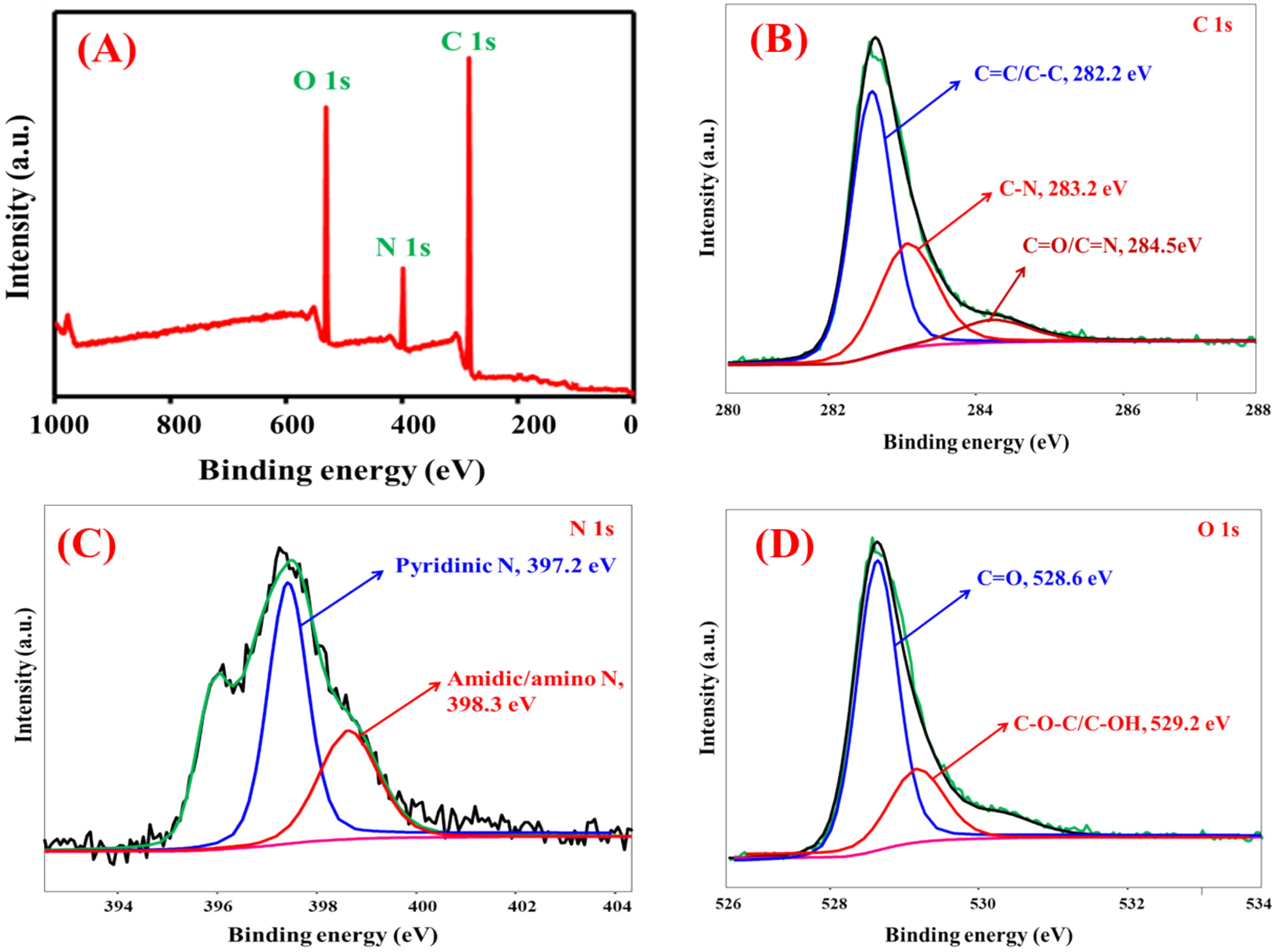

Fig. S1. Full XPS survey (A), C 1s (B), N 1s (C), and O 1s (D) spectra of BG@CDs

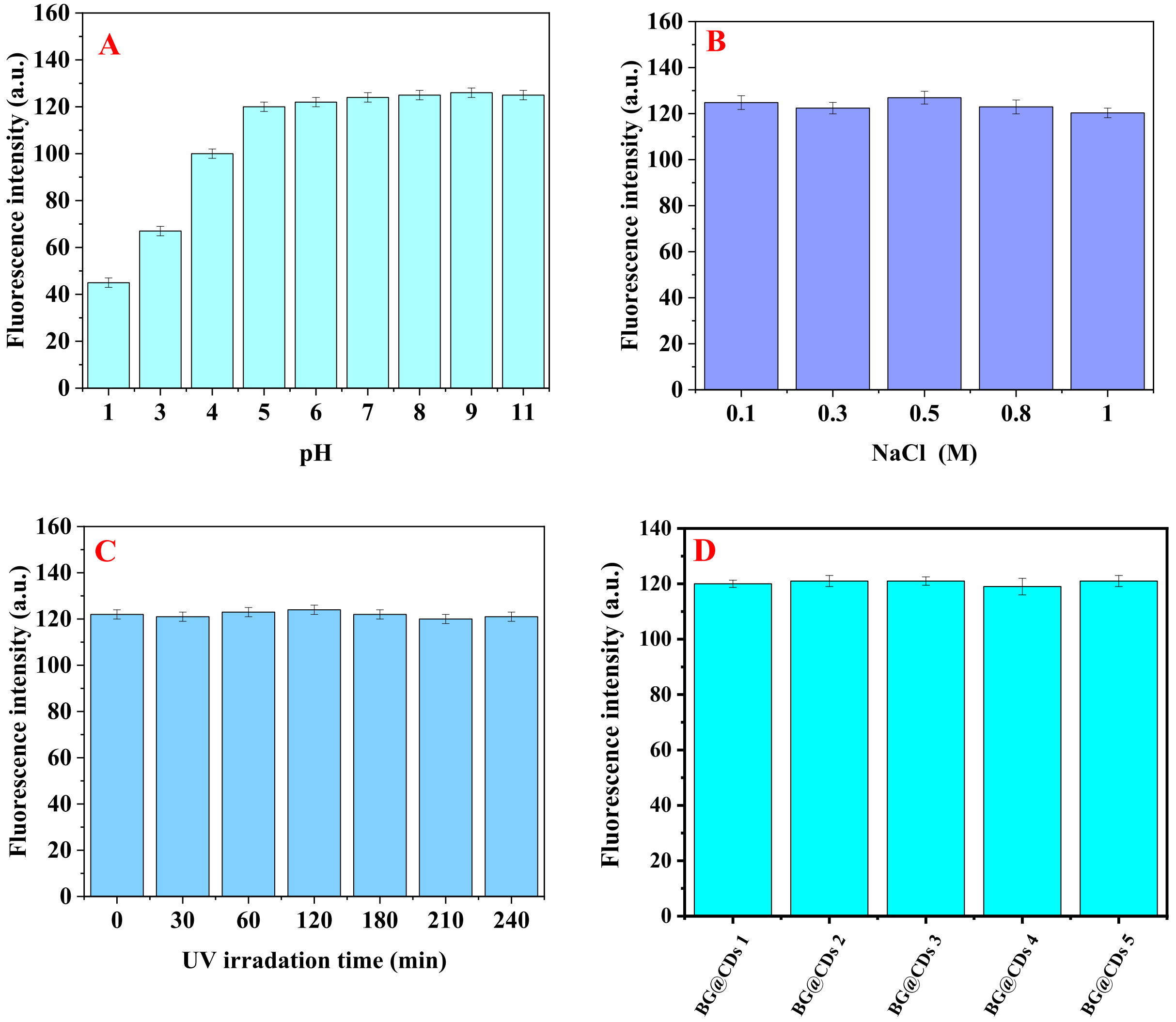

Fig. S2. Effect of (A) pH (1–11), (B) ionic strength (0.1-1.0 M), (C) UV irradiation time (0-240 min) and (D) different patches of black grapes (*Vitis vinifera*) on fluorescence intensity of BG@CDs.

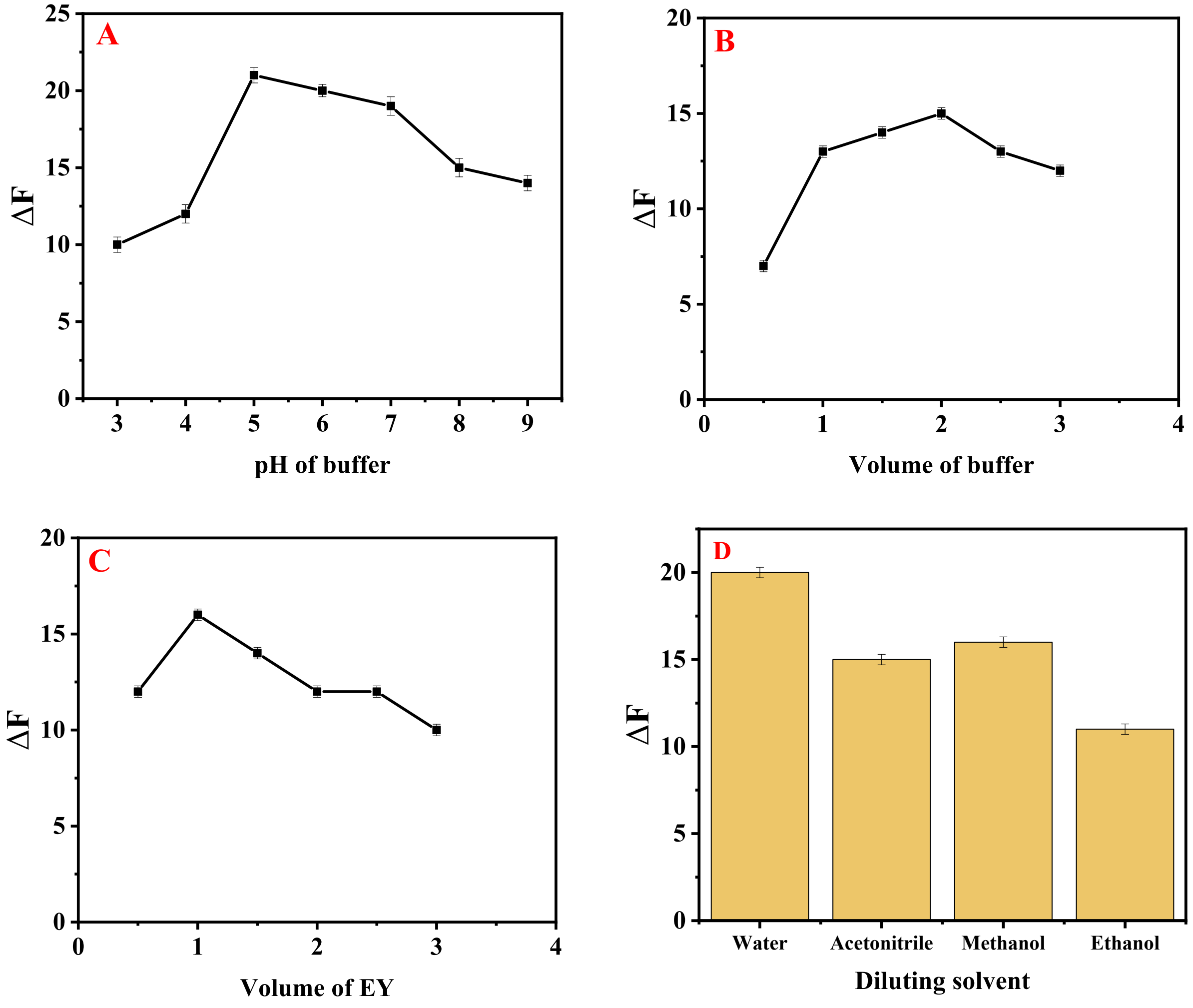

Fig. S3 (A) Effect of buffer pH, (B) Effect of buffer volumes, (C) Effect of eosin Y volumes, and (D) Effect of different diluting solvents on ΔF using 100 ng mL^{-1} of GNT.

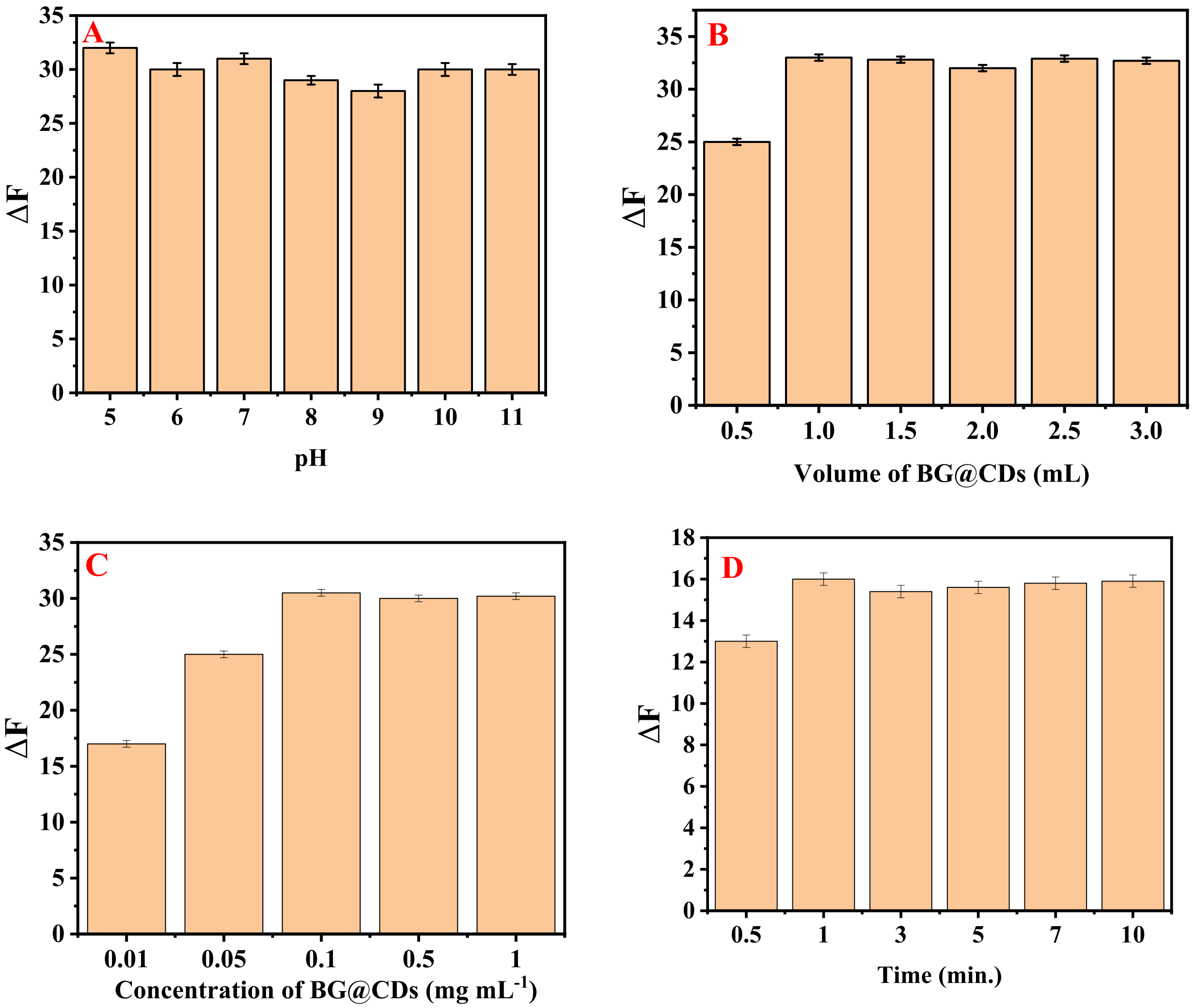

Fig. S4. Influence of the factors affecting ΔF and interactions between BG@CDs and KET (180 ng mL^{-1}). (A) pH (5–11), (B) volume of BG@CDs (0.5-3.0 mL), (C) concentration of BG@CDs (0.01-1.0 mg mL^{-1}), (D) incubation time (0-10 min.).

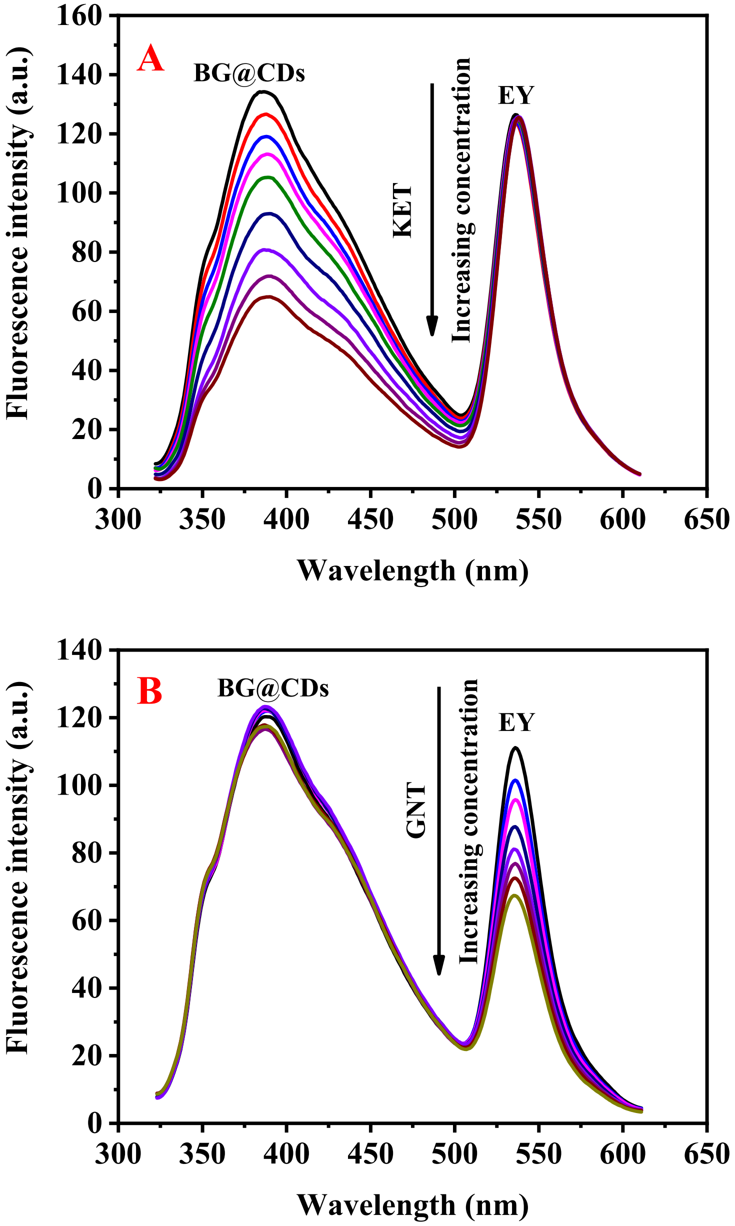

Fig. S5 The influence of different concentrations of (A) KET (20 – 400 ng mL^{-1}) and (B) GNT (50 – 200 ng mL^{-1}) on the fluorescence emission of EY/BG@CDs.

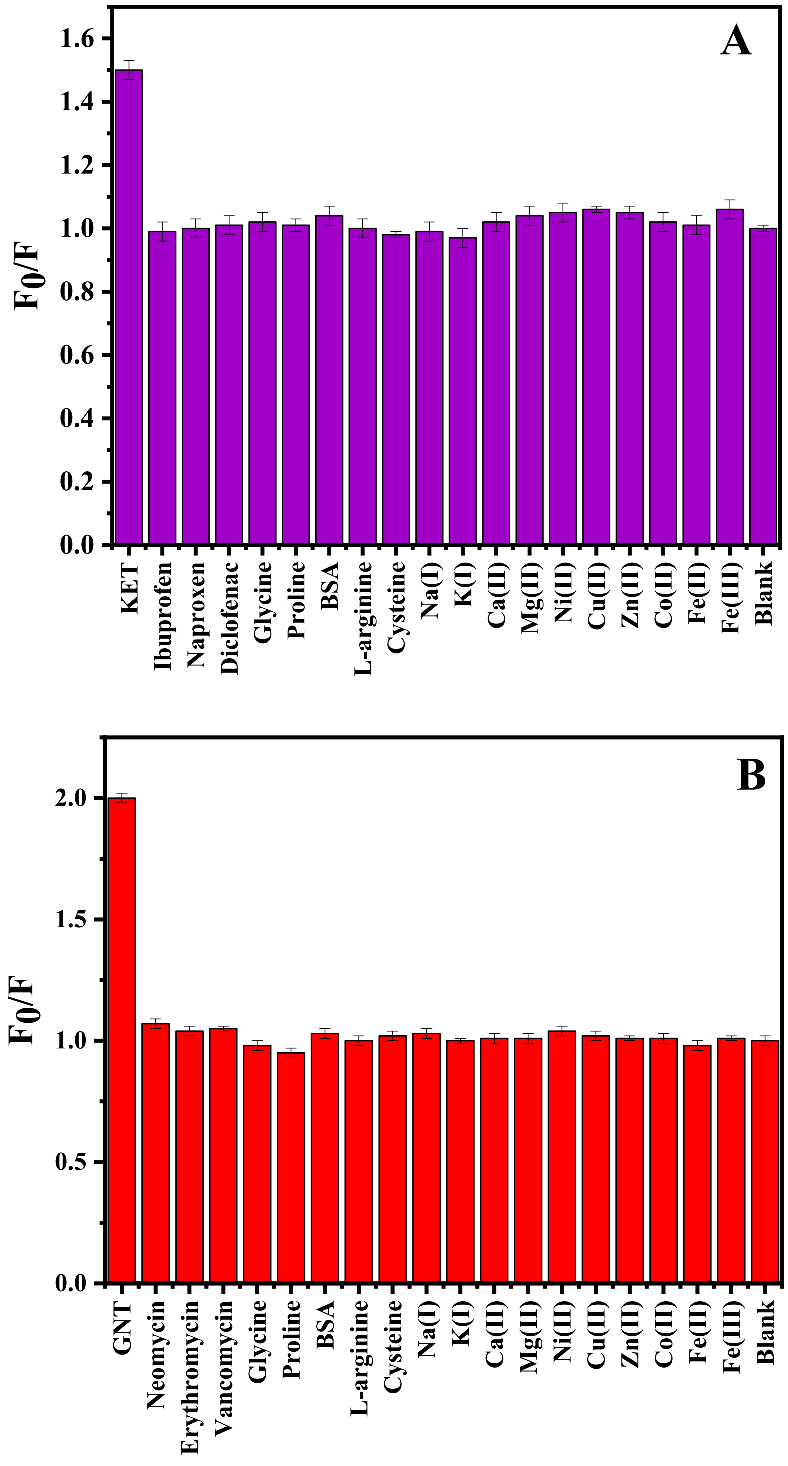

Fig. S6 (A) Relative fluorescence intensity after incorporation of different species to the prepared BG@CDs; (B) The effect of different species on the relative fluorescence intensity of

EY.

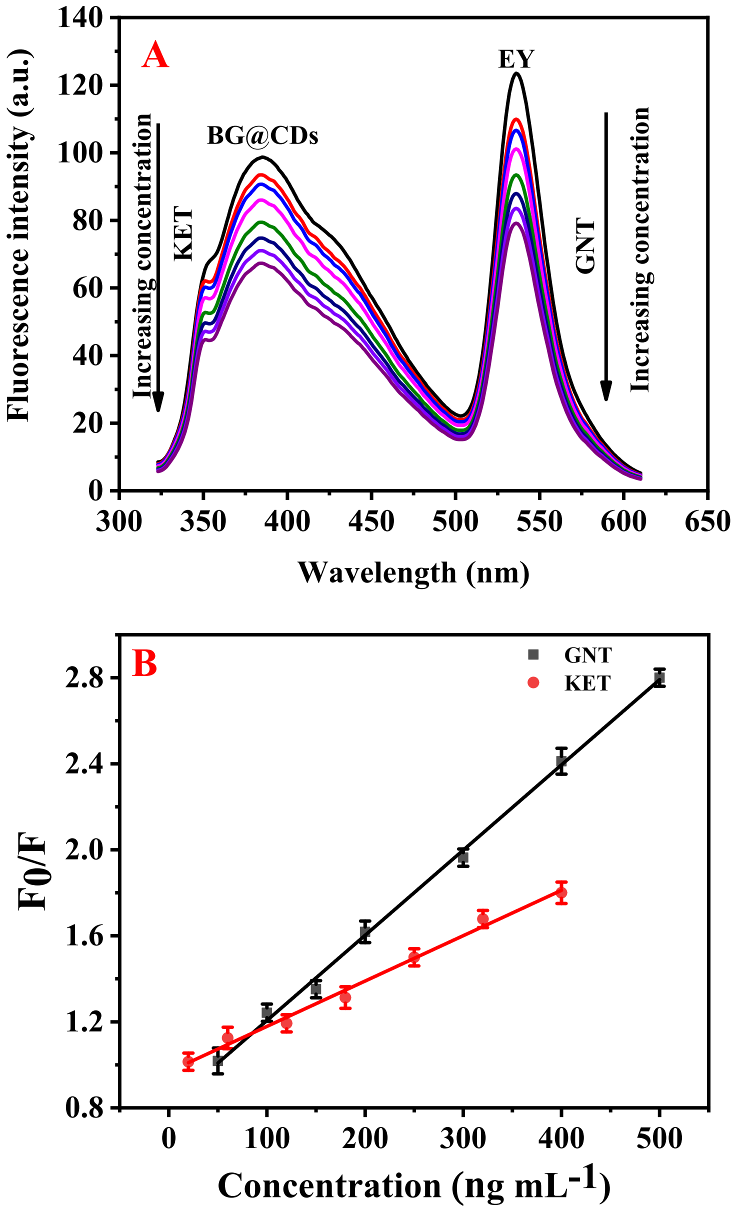

Fig. S7 (A) The influence of different concentrations of GNT (60 – 500 ng mL^{-1}) and KET (25 – 400 ng mL^{-1}) on the fluorescence emission of EY and BG@CDs for their simultaneous determination in spiked rabbit plasma, respectively. (B) Plot of (F_0/F) versus concentration of GNT/KET.
